# Frequency-dependent force direction elucidates neural control of balance

**DOI:** 10.1186/s12984-021-00907-2

**Published:** 2021-09-25

**Authors:** Kaymie Shiozawa, Jongwoo Lee, Marta Russo, Dagmar Sternad, Neville Hogan

**Affiliations:** 1 Department of Mechanical Engineering, Massachusetts Institute of Technology, Cambridge, MA 02139 USA; 2 Department of Neurology, Tor Vergata Polyclinic and Laboratory of Neuromotor Physiology, Santa Lucia Foundation, Rome, Italy; 3 Department of Biology, Northeastern University, Boston, MA 02115 USA; 4 Department of Electrical and Computer Engineering, Northeastern University, Boston, MA 02115 USA; 5 Department of Physics, Northeastern University, Boston, MA 02115 USA; 6 Department of Brain and Cognitive Sciences, Massachusetts Institute of Technology, Cambridge, MA 02139 USA

**Keywords:** Posture and balance, Inverted pendulum models, Ground reaction forces, Neural control, Biomechanics

## Abstract

**Background:**

Maintaining upright posture is an unstable task that requires sophisticated neuro-muscular control. Humans use foot–ground interaction forces, characterized by point of application, magnitude, and direction to manage body accelerations. When analyzing the directions of the ground reaction forces of standing humans in the frequency domain, previous work found a consistent pattern in different frequency bands. To test whether this frequency-dependent behavior provided a distinctive signature of neural control or was a necessary consequence of biomechanics, this study simulated quiet standing and compared the results with human subject data.

**Methods:**

Aiming to develop the simplest competent and neuromechanically justifiable dynamic model that could account for the pattern observed across multiple subjects, we first explored the minimum number of degrees of freedom required for the model. Then, we applied a well-established optimal control method that was parameterized to maximize physiologically-relevant insight to stabilize the balancing model.

**Results:**

If a standing human was modeled as a single inverted pendulum, no controller could reproduce the experimentally observed pattern. The simplest competent model that approximated a standing human was a double inverted pendulum with torque-actuated ankle and hip joints. A range of controller parameters could stabilize this model and reproduce the general trend observed in experimental data; this result seems to indicate a biomechanical constraint and not a consequence of control. However, details of the frequency-dependent pattern varied substantially across tested control parameter values. The set of parameters that best reproduced the human experimental results suggests that the control strategy employed by human subjects to maintain quiet standing was best described by minimal control effort with an emphasis on ankle torque.

**Conclusions:**

The findings suggest that the frequency-dependent pattern of ground reaction forces observed in quiet standing conveys quantitative information about human control strategies. This study’s method might be extended to investigate human neural control strategies in different contexts of balance, such as with an assistive device or in neurologically impaired subjects.

## Background

Controlling balance during standing and walking is a fundamental necessity for human mobility. Although maintaining upright posture involves little overt movement, its inherently unstable nature poses an interesting sensorimotor control problem [1–4].

While many recent studies have investigated balance by applying perturbations to the individual [1, 5–7], it is also important to understand how humans maintain their balance without external perturbations, i.e., during quiet standing. In particular, the center of pressure and the fluctuations of the center of mass have been commonly used to evaluate balance performance during quiet standing [8, 9]. However, studying the center of mass and/or the center of pressure trajectories alone is insufficient to describe the complex dynamics and control of the multi-segmented human body. Insights can be gained by investigating how humans use the direction of their foot–ground interaction force, which is the outcome of a complex sensorimotor control process that involves timed muscle activity, biomechanical constraints, and sensory feedback from multiple pathways. Importantly, the ground reaction forces directly contribute to the centroidal dynamics of the human body [10]. The orientation of the ground reaction force vector and where its line-of-action lies relative to the center of mass may give further insight into how human subjects control the translational motion of the center of mass and net angular motion of the body.

Recently, Gruben and colleagues suggested a new method to study the relation between the orientation of the ground reaction force vector and the center of pressure in human subjects during quiet standing [11, 12]. Net ground reaction forces at different times, which have different orientations and points of application (centers of pressure), intersect at some point in space. The authors defined this point as the intersection point and examined its relation to the center of mass of the standing individual. Because the height of the intersection point relative to the center of mass determines the translational and angular components of centroidal accelerations, it provides a compact geometric representation that is useful for understanding the dynamics and control of human standing balance. When analyzing the force vectors in the frequency domain, this previous study [11] found that the vertical position of the intersection point exhibited a consistent pattern across subjects. With this observation, the authors suggested that the frequency-dependent intersection point characterizes the neural controller of human balance. However, the biomechanics of upright posture might account for some of the variation of intersection point height across different frequency bands. Therefore, this study aimed to elucidate the extent to which the frequency-dependent variation of the intersection point could be attributed to neural control strategies or to biomechanics.

To this end, the first objective was to develop the simplest competent and neuromechanically justifiable dynamic model that could account for the consistent pattern observed across multiple subjects [11]. Second, we examined the hypothesis that the neural control strategies in standing balance would economize control effort [6]. To test this hypothesis, we took advantage of the linear quadratic regulator (LQR) [13], a well-established optimal control method, that enabled a systematic search of physiologically-plausible controller parameters [2, 14–16].

## Methods

### Human experiment

#### Experimental procedure

In the previous study [11], ten unimpaired, young participants (24.2 ± 10.3 years) were asked to stand quietly while viewing a mark at head height 1 m away. Each participant completed one 50 s trial standing on a 6-axis force-plate measuring at 1000 Hz. The analysis was confined to the sagittal plane. The subjects’ average mass and height were 71 kg and 1.75 m, respectively.

#### Intersection point

**Fig. 1:**
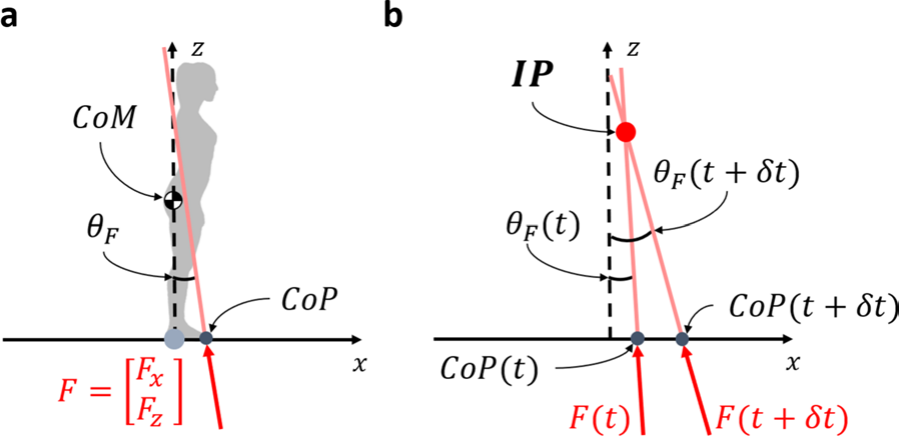
**a** Net ground reaction force,* F*, made up of horizontal and vertical components, $F_x$ and $F_z$, acts at the center of pressure,* CoP*, and has an orientation, $\theta _F$. The center of mass,* CoM*, is also shown. **b** Two force vectors from two different time points, which are defined by their respective $\theta _F$ and* CoP*, intersect at the intersection point, IP

The intersection point was defined as a point in space where the net ground reaction force vectors at adjacent time-steps intersect [11], as illustrated in Fig. 1. The intersection point is a geometric representation of the relation between the ground reaction force and the center of pressure. This point was originally identified with the goal to understand how humans maintain balance during walking [17, 18]; Gruben and colleagues were the first to apply it to understand the mechanics of standing balance [11].

Assuming subtle movements of the body and small variations in ground reaction force orientations, the orientation of the ground reaction force ($\theta _F$) can be approximated as

1 $$\begin{aligned} -\frac{F_x}{F_z} = \tan \theta _F \approx \theta _F, \end{aligned}$$

where $F_x$ and $F_z$ are the horizontal and vertical components of the net ground reaction force, respectively.

The height of the intersection point (*IP*) of two forces at adjacent times ($F(t), F(t+\delta t)$) is

$$\begin{aligned} IP (t)= \frac{{{{CoP}}}(t) - {{{CoP}}}(t+\delta t)}{\theta _{F}(t) - \theta _{F}(t+\delta t)}, \end{aligned}$$

where* CoP*(*t*) is the center of pressure at time *t*.

#### Frequency-dependence of the intersection point

**Fig. 2:**
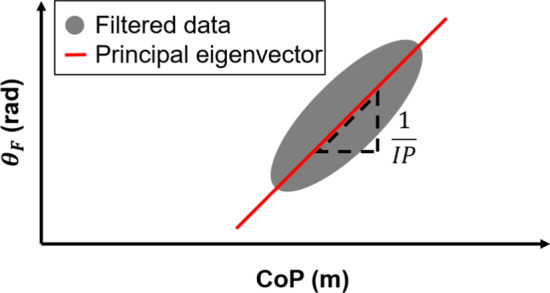
Relation between $\theta _F$ and* CoP* for one simulation trial. The data were processed by filtering the* CoP* and $\theta _F$ signals using a 2nd-order bandpass filter with a 0.2 Hz wide frequency band. The principal eigenvector of the covariance matrix of the filtered data was extracted. The intersection point (*IP*) was computed as the inverse of the angle of the principal eigenvector. Note that the time series of the data was approximated as an ellipse in this schematic illustration

Investigating the system response in the frequency domain often yields insight into the structure of a dynamic system. To parse the time series into frequency bands, a Hamming window with the length of the entire data set was first applied to both $\theta _F$ and* CoP* signals. $\theta _F$ and* CoP* signals were then bandpass-filtered (zero-lag, 2nd-order Butterworth) and parsed into bands of 0.2 Hz width centered on frequencies from 0.5 to 7.9 Hz (38 nominally non-overlapping bands). Finally, the principal eigenvector of the best-fit covariance matrix of $\theta _F$ plotted against* CoP* (both signals detrended to have zero-mean) was extracted for each band. Its slope is equivalent to the inverse of the intersection point, as illustrated in Fig. 2. Assuming small variation between the forces,

$$\begin{aligned} \begin{aligned} \theta _{F}(t+\delta t)&\approx \theta _{F}(t) + \delta \theta _F(t),\\ {{CoP}}(t+\delta t)&\approx  {CoP}(t) + \delta {{CoP}} (t), \end{aligned} \end{aligned}$$

the lower-order component of the intersection point height (*IP*) can be approximated as

2 $$\begin{aligned} IP \approx \frac{d{CoP}}{d\theta _F}, \end{aligned}$$

and re-arranging (2) results in

3 $$\begin{aligned} d\theta _F = \frac{1}{IP}d{CoP}. \end{aligned}$$

Gruben and colleagues [11] found that the vertical position of the intersection point exhibited a consistent pattern across subjects: it was above the center of mass at low frequencies and decreased as frequency increased, reaching an asymptote below the center of mass at higher frequencies as shown in Fig. 4a in the  Results section.

### Simulation

#### Single inverted pendulum model

We first investigated whether a single inverted pendulum, which is a widely accepted model for human quiet standing [4], could reproduce the experimental observations. Theoretical analysis showed that the model could not adequately reproduce the experimental observation in [11], because the intersection point height of the single inverted pendulum was always above the center of mass (Appendix 1). Hence, a multi-degree-of-freedom model was required.

**Fig. 3:**
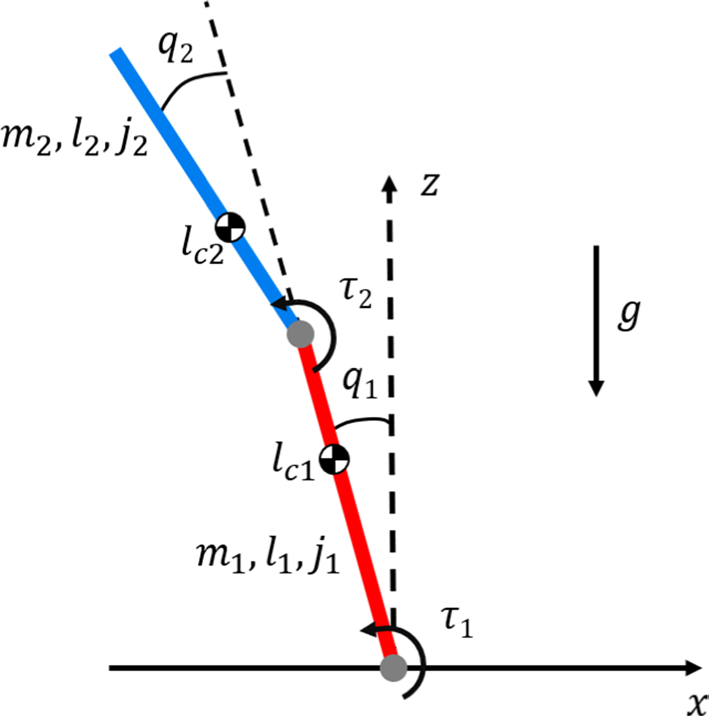
Double inverted pendulum model with angle ($q_i$) and torque ($\tau _i$) conventions and parameter values for mass ($m_i$), length ($l_i$), center of mass ($l_{ci}$), and moment of inertia about the center of mass height ($j_i$) for each link* i*. The direction of gravity (*g*) is also defined

#### Double inverted pendulum model

The double inverted pendulum model that was used to simulate a multi-segmented human body is illustrated in Fig. 3. The lumped model parameters summarized in Table 1 used the anthropometric distribution of male subjects in the sagittal plane [19] based on the average height and weight of the subjects from [11]. Any mass and length below the ankle was neglected, as the simulation assumed the ankle to be a pin joint. The center of mass positions were measured with respect to the ankle joint for link 1 and the hip joint for link 2. The moments of inertia were calculated about the center of mass of each link.

**Table 1:** Lumped model parameters

Symbol	Parameter (units)	Value	
		Link 1 Lower body	Link 2 Upper body
*m*	Mass (kg)	26.30	42.88
*l*	Length (m)	0.867	0.851
$l_c$	Center of mass height (m)	0.589	0.332
*j*	Moment of inertia ($\hbox {kgm}^2$)	1.400	2.227
*g*	Gravitational acceleration (m/$\hbox {s}^2$)	9.81	

The equations of motion of the double inverted pendulum were

4 $${\mathbf{M}}({\mathbf{q}})\ddot{{\mathbf{q}}}+{\mathbf{C}}({\mathbf{q}},\dot{{\mathbf{q}}})\dot{{\mathbf{q}}}+{\mathbf{G}}({\mathbf{q}})= \varvec{\tau}$$

where ${\mathbf {M}}({\mathbf {q}}) \in {\mathbb {R}}^{2\times 2}$ is the inertia matrix, ${\mathbf {C}}({\mathbf {q}},\dot{\mathbf{q}}) \in {\mathbb {R}}^{2\times 2}$ contains the Coriolis and centrifugal terms, ${\mathbf {G}}({\mathbf {q}}) \in {\mathbb {R}}^{2\times 1}$ are gravitational torques, and ${\varvec{\tau }}=[\tau _1, \tau _2]^T \in {\mathbb {R}}^{2\times 1}$ is the joint torque vector (see Appendix 2 for full symbolic inertia, centrifugal, and gravitational matrices). Generalized coordinates are ${\mathbf {q}} = [q_1, q_2]^T \in {\mathbb {R}}^{2\times 1}$ as defined in Fig. 3. These variables represent the sagittal plane angular displacements of the ankle and hip joints, respectively.

Defining the state vector as ${\mathbf {x}} = [{\mathbf {q}}^T, \dot{\mathbf{q}}^T]^T$, (4) can be rewritten in state-determined form as

5 $${\dot{\mathbf{x}}} = \left[ {\begin{array}{*{20}c} {{\dot{\mathbf{q}}}} \\ { - {\mathbf{M}}({\mathbf{q}})^{{ - 1}} ({\mathbf{C}}({\mathbf{q}},{\dot{\mathbf{q}}}){\dot{\mathbf{q}}} + {\mathbf{G}}({\mathbf{q}})) + \varvec{\tau }} \\ \end{array} } \right]. $$

The internal perturbations that cause persistent sway in quiet standing were simulated by additive noise

6 $$\begin{aligned} {\varvec{\tau }}={\varvec{\tau }}_{\mathrm{{ctl}}}+{\mathbf {w}}, \end{aligned}$$

where ${\varvec{\tau }}_{\mathrm{{ctl}}} = [\tau _{\mathrm{{ctl}},1},\tau _{\mathrm{{ctl}},2}]^T$ are the ankle and hip torques that stabilize the body. In this study, we assumed the noise ${\mathbf {w}} \in {\mathbb {R}}^{2\times 1}$ was white, mutually uncorrelated, and that it followed a zero-mean Gaussian distribution with covariance matrix $E\{\mathbf {ww}^T\}=diag\{\sigma _1^2, \sigma _2^2\}$. The relative strength of the two noise sources was defined as $\sigma _r =\sigma _1/\sigma _2$, where $\sigma _1$ and $\sigma _2$ are the noise at the ankle and hip, respectively.

$F_x$ and $F_z$, the horizontal and vertical components of the ground reaction force, were computed as follows

$$\begin{aligned} F_x = m\ddot{r}_{CoM,x}, \quad F_z = m(\ddot{r}_{CoM,z}+g), \end{aligned}$$

where $m=m_1+m_2$ is the total mass of the body, $\ddot{r}_{CoM,x}$ and $\ddot{r}_{CoM,z}$ are the horizontal and vertical components of the center of mass acceleration. The center of pressure was then computed as

7 $$\begin{aligned} {{CoP }} = \frac{\tau _1}{F_z}. \end{aligned}$$

#### Linear quadratic regulator

This study used a nonlinear model with a linear controller. Hence, the nonlinear equations of motion (5) were first linearized about the upright balancing posture at rest (${\mathbf {x}}_* = {\mathbf {0}}$ and ${\varvec{\tau }}_* = {\mathbf {0}}$) as follows

8 $$\begin{aligned} \dot{\bar{{\mathbf {x}}}} = {\mathbf {A}}_{lin}\bar{{\mathbf {x}}} + {\mathbf {B}}_{lin}{\varvec{{\bar{\tau }}}} + {\mathbf {B}}_{lin}{\mathbf {w}}, \end{aligned}$$

where $\bar{{\mathbf {x}}} = {\mathbf {x}} - {\mathbf {x}}_*$, ${\varvec{{\bar{\tau }}}} = {\varvec{\tau }}_{\mathrm{{ctl}}} - {\varvec{\tau }}_*$, and ${\mathbf {A}}_{lin}$ and ${\mathbf {B}}_{lin}$ are linearized state and input matrices, respectively (see Appendix 3 for the linearized state-space matrices).

As normal human standing is evidently stable in the upright position, the LQR method was chosen as it guarantees a stable closed-loop system^1^. The LQR is an optimal linear state-feedback controller that minimizes the quadratic cost function

9 $$ J = \int_{0}^{\infty}[\bar{\mathbf{x}}^T(t)\mathbf{Q}\bar{\mathbf{x}}(t) + \varvec{\bar{\tau}}^T(t)\mathbf{R}\varvec{\bar{\tau}}(t)]dt$$

to determine control torques

10 $$\begin{aligned} {\varvec{\tau }}_{\mathrm{{ctl}}} = -{\mathbf {K}}_{LQR}{\mathbf {x}}, \end{aligned}$$

where ${\mathbf {K}}_{LQR}$ is the optimal control gain matrix found via the LQR procedure. The matrices ${\mathbf {Q}}$ and ${\mathbf {R}}$ in (9) weight the state and input deviations from zero.

We parameterized the input weighting matrix as

11 $$\begin{aligned} {\mathbf {R}} = \alpha \begin{bmatrix} \beta &{}0 \\ 0 &{} 1/\beta \end{bmatrix}, \end{aligned}$$

to facilitate exploration of two important features: the relative cost between state deviation and control effort, determined by the parameter $\alpha$, and the relative magnitude of hip and ankle effort, determined by the parameter $\beta$.

When $\alpha$ is large, control effort is reduced to a minimum value required for stability. Thus, with this choice of $\alpha$, the need to add joint torque limits to the model was eliminated. Additionally, with large $\alpha$, the resulting closed-loop system has a well-defined behavior (placing its poles at the mirror images of the unstable open-loop poles) that is independent of the state weighting matrix ${\mathbf {Q}}$. To evaluate the working hypothesis that humans economize effort, the minimal-effort solution was of interest. Consequently, the choice of the state weighting matrix was not critical, and ${\mathbf {Q}} = I_4$, the identity matrix with dimension 4, was chosen to equally penalize each state’s deviation from equilibrium.

When $\beta > 1$, the ankle torque is penalized more heavily than the hip, and vice versa when $\beta < 1$. Since the LQR controller minimizes a quadratic cost function (9) to achieve stability, only the symmetric components of ${\mathbf {R}}$ affect the result. The diagonal values of ${\mathbf {R}}$ were selected such that the size of the matrix (i.e., the product of its eigenvalues) was always equal to 1 and only the components’ ratio affected the results. This choice of parameters also allowed for conclusions to be drawn about the relative penalty on the ankle and hip joints.

#### Simulation protocol

The simulation was conducted using semi-implicit Euler integration. The initial condition was set to ${\mathbf {x}}_0 = [0, 0, 0, 0]^T$. Replicating the experimental protocol of [11], each simulation was run for 50 s at 1000 Hz. All simulations were conducted in MATLAB 2020a (Mathworks, Natick MA).

To observe the effect of altering the LQR parameters on the frequency-dependence of the intersection point and to find the simplest model that could reproduce the human data, various parameters were tested using the following procedure. First, the parameter that weights the relative cost of the control input, $\alpha$, was set to a large value to ensure minimal control ($\alpha > 10^4$). This design choice effectively reduced the number of parameters to two ($\beta$ and $\sigma _r$) as the essential intersection point frequency-dependence (above the center of mass at low frequencies, below at high frequencies) varied little when $\alpha$ was sufficiently large. Then the noise ratio, $\sigma _r$, was adjusted to produce the best fit at high frequencies while setting $\beta = 1$. Lastly, $\beta$ was varied to produce the best fit in the frequency range where the intersection point height was approximately equal to the center of mass height. At the same time, it was ensured that the asymptotic behavior and the fit at high frequencies were maintained.

40 trials were conducted for each tested parameter set to enable statistical analysis of the simulated dependence of the intersection point height on frequency.

**Fig. 4:**
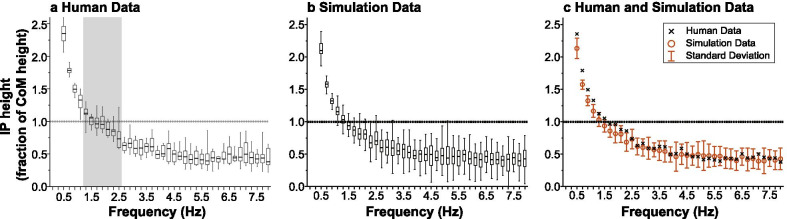
Comparison of the intersection point’s frequency-dependence from: **a** Human experimental data (reproduced from [11] with permission) and **b** Simulation data with best-fit parameters. **c** The mean of the best-fit simulation data overlaid on the median of the human data from [11]. Within the frequency band from 1.2 to 2.6 Hz for the human data, there was no significant difference (with 95% confidence) between the mean of the intersection point height and the center of mass height. This frequency band is marked by the shaded region. The high-frequency asymptote (3–8 Hz range) of the intersection point was $0.479 \pm 0.028$ and $0.468 \pm 0.021$ for the human and simulation data, respectively (with 95% confidence)

### Comparison of simulation and human experimental results

When determining the goodness of fit across different model parameter conditions, the average difference of the simulated data and the human subject data from [11] was computed over selected frequency bands by

$$ \begin{aligned} &  {\text{Average difference}}\\ \quad &= \frac{\sum_{i=1}^{N} {\text{Human Data}}_i - {\text{Simulation Data}}_i}{N_{band}},\\ & \quad i = 1, 2, \ldots N_{band}. \end{aligned}$$

where ${\text {Human Data}}_i$ is the median of the intersection point height as a fraction of the center of mass height reported by [11] at each frequency band; $\text {Simulation Data}_i$ is the average intersection point height as a fraction of the center of mass height across 40 trials of the simulation data in a given frequency band; $N_{band}$ is the number of frequency bands for which the difference in the data was computed. Because balance is characterized by only small motions, a constant center of mass height was assumed.

To identify the onset of the high-frequency asymptote, the human data were fit to an exponential function. The best-fit decay constant was $T\cong 1$ Hz. Assuming the curve reached its asymptote at frequency $\cong 3T$, the asymptote started at 3 Hz. Therefore, the difference between the simulated and experimental asymptote was evaluated at frequencies from 3 to 8 Hz ($N_{band} = 25$). To evaluate the effect of different controller parameters on the frequency range in which the experimentally observed intersection point height crossed the center of mass height, the average difference between simulation and human data was evaluated at frequencies from 1.2 to 2.6 Hz ($N_{band} = 7$). This range encompassed the frequencies in which the observed intersection point height was not statistically different from the center of mass height in [11]. One-sample t-tests were used to evaluate the difference between the center of mass height and the simulated mean intersection point height. The 95% confidence interval of the mean of the difference was computed as well.

## Results

### Minimum required model complexity

Theoretical analysis showed that a single-degree-of-freedom inverted pendulum model could not reproduce the experimental observation in [11]. The intersection point height of a single inverted pendulum model was always above the center of mass for any selection of parameters (Appendix 1). Hence, we proceeded with a double inverted pendulum, i.e. with two degrees of freedom, to approximate the multi-segmented human body.

### Best-fit model parameter set

The simulated center of mass height did not deviate far from 0.97 m, the height of the center of mass when perfectly upright, justifying the assumption of small angular displacement. In what follows, the center of mass height was therefore assumed to be constant.

The simulated frequency-dependent intersection point response for the parameter set, $\alpha = 10^6$, $\beta = 0.3$, $\sigma _r = 0.9$, best matched the human subject data from [11] as shown in Fig. 4. Both simulations and human experimental results show that the intersection point height crossed the center of mass height in similar frequency bands (1.2–2.6 Hz) and had similar asymptotes at higher frequencies. The difference compared to human data for this parameter set was $0.101 \pm 0.040$ in the 1.2–2.6 Hz range and $0.011 \pm 0.019$ in the 3–8 Hz range (both within the 95% confidence interval). While the average mass and height in our simulations were taken from [11] to afford best comparison with the human data in Fig. 4c, varying the mass and height parameters over physiologically plausible values did not significantly affect the results.

### Varying model parameters

**Fig. 5:**
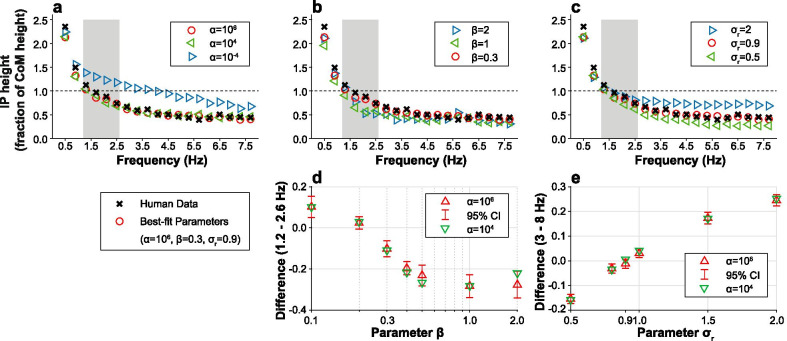
Effect of varying parameter values on the frequency-dependence of the intersection point. Each model parameter was varied with respect to the “best-fit” parameter set that closely resembled human subject data observed in [11] ($\alpha = 10^6$, $\beta = 0.3$, $\sigma _r = 0.9$). The height of the center of mass is indicated by a dashed line. The shaded region, based on human experiments, indicates the frequency band in which the mean of the intersection point height was not significantly different from the center of mass height in [11]. **a** The parameter $\alpha$ determined the cost of the overall magnitude of the control effort relative to state deviation from equilibrium. When varying $\alpha$, the other parameters were set to $\beta = 0.3$ and $\sigma _r = 0.9$. **b** The parameter $\beta$ determined the relative cost of ankle and hip torque. When $\beta > 1$, there was more penalty on ankle torque. When varying $\beta$, the other parameters were set to $\alpha = 10^6$ and $\sigma _r = 0.9$. **c** The parameter $\sigma _r$ determined the relative strength of noise in the ankle and the hip. When $\sigma _r > 1$, ankle noise was greater than hip noise. When varying $\sigma _r$, the other parameters were set to $\alpha = 10^6$ and $\beta = 0.3$. **d** The difference of the intersection point in the 1.2–2.6 Hz frequency range of the simulated data compared to the human subject data [11] with respect to $\beta$. The parameter $\sigma _r$ was kept at 0.9. **e** The difference of the intersection point in the 3–8 Hz frequency range of the simulated data compared to the human subject data [11] with respect to $\sigma _r$. The parameter $\beta$ was kept at 0.3. In both cases, the effect of varying $\alpha$ is also shown. The error bars indicate the 95% confidence interval of the mean of difference when $\alpha = 10^6$

Varying the simulation parameters affected both the frequency at which the intersection point crossed the height of the center of mass and the high-frequency asymptote. The effect of changing parameter values is presented in Fig. 5a–c. The differences between simulation and human data for certain parameter sets are shown in Fig. 5d, e.

#### Effect of $\alpha$

As shown in Fig. 5a, when $\alpha$, the weighting of control effort relative to state deviation, was increased, the intersection point crossed the center of mass at lower frequencies. For example, when $\alpha$ was varied from $10^{-4}$ to $10^6$, the frequency at which the intersection point crossed over the center of mass moved from 3.9 to 1.5 Hz. As expected from theory, when $\alpha$ was relatively large ($\alpha > 10^4$), there was little effect of varying its value on the difference between human and simulation data for different model parameter sets, as shown in Fig. 5d, e.

#### Effect of $\beta$

As shown in Fig. 5b, when $\beta$ was decreased, i.e. when hip control was penalized more than ankle control, the intersection point crossed the center of mass at higher frequencies. For example, when $\beta$ was varied from 1 to 0.3, the frequency at which the intersection point crossed over the center of mass moved from 1.1 to 1.5 Hz. In Fig. 5d, $\beta = 0.2$ was shown to be the parameter with the smallest difference (0.024) in the 1.2–2.6 Hz range when $\alpha = 10^6$. However, both the selection of $\beta = 0.2$ and $\beta = 0.1$ sacrificed the high-frequency fit, increasing the absolute value of the difference in that range by 0.102 and 0.292, respectively, compared to $\beta = 0.3$ when $\alpha = 10^6$. As $\beta$ deviated from $\beta = 0.3$, the absolute value of the difference in the 1.2–2.6 Hz range increased by 0.181 when $\beta = 1$ and $\alpha = 10^6$.

#### Effect of $\sigma _r$

Adjusting $\sigma _r$ shifted the high frequency asymptote (3–8 Hz) of the intersection point, as shown in Fig. 5c. Variation of the high-frequency asymptote of the intersection point height was predicted by the analysis presented in Appendix 4. Here, the two extremes, zero noise in the ankle ($\sigma _r=0$) and the hip ($\sigma _r=\infty$), provided lower and upper bounds for the high frequency asymptote. When compared to the best-fit height of the intersection point at high frequencies, the asymptote was 55% higher when $\sigma _r = 2$ (more noise in the ankle) and 30% lower when $\sigma _r = 0.5$ (more noise in the hip). In Fig. 5e, $\sigma _r = 0.9$ is shown to be the best-fit parameter with the smallest difference value, at −0.011 when $\alpha = 10^6$. As $\sigma _r$ deviated from the best-fit value, the difference increased to 0.245 when $\sigma _r = 2$ and to −0.154 when $\sigma _r = 0.5$, when $\alpha = 10^6$.

## Discussion

This study analyzed a deliberately simplified model of human quiet standing with a stabilizing linear optimal controller to better understand the origin of the frequency-dependent intersection point reported by Gruben and colleagues [11].

The simplest competent model required two degrees of freedom (ankle and hip) with a stabilizing controller that used minimal control effort and more ankle torque than hip torque. We successfully identified a narrow range of parameters that provided not only a quantitative reproduction of experimental observations, but also qualitative insight.

### Neural control or biomechanics?

Despite the biomechanical constraints that limit the admissible center of mass accelerations and the centers of pressure [14, 20], the ground reaction forces that comply with these constraints are infinite [17, 21]. Beyond the obvious fact that the musculo-skeletal system is inherently unstable without a neural controller, we should not expect mechanics alone to determine the intersection point’s frequency dependence. When Gruben and colleagues [11] analyzed the frequency dependence of the intersection point, they observed a consistent trend across multiple subjects and suggested that this consistency was the signature of a neural controller employed by humans during balance.

The results of our simulations replicated the frequency dependence of the intersection point reported for human standing in the sagittal plane—the intersection point was above the center of mass at low frequencies and below the center of mass at high frequencies, as shown in Fig. 4. To understand the general frequency-dependent trend of the intersection point, first consider an extreme case at very low frequencies where the two-degree-of-freedom pendulum behaves similar to a single rigid body: its intersection point would be above the center of mass, like that of the single inverted pendulum (Appendix 1). A double inverted pendulum can also be stabilized solely by hip torque, i.e. zero ankle torque. In the latter case, the system would exhibit higher frequency behavior while maintaining the intersection point height to be zero (from (7) and (2) with $\tau _1=0$). This indicates that the general trend for the intersection point to be above the center of mass at low frequencies and below at high frequencies may be a consequence of biomechanics, i.e. a double inverted pendulum stabilized about upright posture.

However, biomechanics cannot account for the specific details of the frequency variation of the intersection point height. Somewhere between the low-frequency and high-frequency regimes, the intersection point must cross from above to below the center of mass height; this particular crossing point is not specified by biomechanics. Similarly, biomechanics does not dictate the asymptote to which the intersection point height converges at high frequencies. In fact, both the intersection point’s asymptote and the frequency at which the intersection point height crossed that of the center of mass varied substantially across the tested parameter values. It was only a small set of model parameters that could replicate human behavior. Therefore, we conclude that the details of the profile of intersection point height with frequency reflect a neural control strategy used by humans during quiet stance.

### Physiologically-plausible best-fit parameters

The main contribution of this work is to deploy a deliberately-simplified mathematical analysis to elucidate how experimental observations of the frequency-dependence of the intersection point may inform the neural control of balance. To conduct this quantitative analysis, the model parameters were systematically varied such that the simulated response of the intersection point's frequency-dependence closely replicated human data. To facilitate analysis, we took advantage of the LQR procedure and its well-known properties.

Selecting $\alpha = 10^6$ yielded the best-fit result compared to human data, suggesting that a double inverted pendulum model with minimal control effort can successfully account for the frequency-dependent intersection point observed in humans. Though it is widely assumed that humans generally minimize effort, supporting evidence during quiet standing has been sparse. Our results show that the observed variation of intersection point height with frequency implies that humans minimize control effort rather than reduce state deviation during quiet standing. This is consistent with the conclusion of a previous study reporting that the nervous system does not exert more control effort than necessary to stabilize upright balance [6].

Long transmission delays in the neural system pose a risk to stability of the balance controller. To account for this, the continuous feedback loop gain must be effectively zero at high frequencies regardless of variations in other model parameters. However, muscle mechanical impedance is not limited in this way; it can respond essentially instantaneously. Behavior in the high frequency range is therefore not likely to depend on neural feedback (defined by $\alpha$ and $\beta$), but instead on neuromuscular impedance and noise (defined by $\sigma _r$). Hence, the noise ratio, $\sigma _r$, was adjusted to fit the high-frequency range before fitting the low-frequency range with $\beta$.

Altering the relative noise magnitude in the ankle and the hip torques ($\sigma _r$) shifted the high-frequency asymptote of the intersection point height. The simulation result most similar to human experimental data had a 0.9:1 ankle-to-hip noise ratio.

The $\beta$ value that best described human data in [11] was 0.3 that penalized hip control effort more than ankle control effort. That is, the system is more likely to use the ankle to maintain upright posture than the hip. This is consistent with previous findings that humans primarily use the “ankle strategy” during quiet standing [14, 22–24].

### Single vs. multi-joint model

The observed trend that the intersection point varied with frequency in humans requires multi-segment mechanics (Appendix 1). Although the single inverted pendulum model has been widely used to model quiet human standing [1, 4, 26–29], our finding that a single-segment model cannot adequately describe quiet standing is also consistent with recent literature [6, 30–33].

Why no more than two degrees of freedom? It is patently obvious that the standing human body has many more degrees of freedom. However, although adding a knee joint [34] or multiple segments of the spine might more accurately replicate human biomechanics, it is unclear whether this would improve the insight to be gleaned from experimental observations. In fact, as shown in Appendix 4, the two-segment model yields a high-frequency asymptote for the intersection point height that must lie between zero (corresponding to zero noise at the ankle) and below the center of mass height (corresponding to zero noise at the hip). These two extremes bracket the experimental observations reported in [11]. Thus, the two-segment model used in this study was the simplest that could competently reproduce the experimental results observed in [11].

### Intersection point: a target variable of control or an emergent consequence?

In this study, the feedback signal was the state error (joint angles and velocities) rather than the intersection point height. Even so, the control model was able to replicate the frequency dependence of the intersection point found in humans. Hence, it appears that the intersection point may be an emergent consequence of stabilization rather than a variable explicitly regulated by the controller. Consistent with this hypothesis, a previous study suggested that the force direction pattern observed in human walking might be an emergent property, rather than a target variable of control [35]. However, further experimentation would be required to test this hypothesis.

### Limitations

The simulations conducted in this study assumed simple mechanics. The joint torques in the model are net joint torques that summarize the contributions of various elements, from passive muscle properties to complex neural control. Known features of neuromuscular physiology such as muscle mechanical impedance, neural transmission delay, or sensory noise were omitted. While these features are unquestionably present, our goal was to identify the simplest model competent to reproduce experimental observations. Despite the lack of muscle- and nerve-level detail, our simulations were able to articulate subtle differences between control parameters that influence the frequency-dependence of the intersection point. Nevertheless, including those neurophysiological features might yield further insight; that is deferred to future work.

This study employed a linear full-state feedback controller with a constant gain matrix (proportional feedback of angle and angular velocity) even though the central nervous system comprises many nonlinear neural elements. This decision was motivated by the observation that the body generates only small motions about the upright posture, justifying the use of a linearized model to obtain feedback controller gains. This observation also justified the choice of additive noise, as higher-order terms that characterize nonlinear noise processes are negligible. We therefore modeled the noise as white. However, some studies have indicated that biological noise may be better described by ‘pink’ or Brownian noise [25]. Since the low-pass filter property of inertial mechanics suppresses high-frequency components of the spectrum, this noise model proved to be a convenient and viable option.

Finally, the model employed in this study assumed a perfect state estimator. Future studies might assess the effect of including sensory information into the motor controller by employing other control architectures, for instance, using an adaptive [5] or optimal state estimator [14] instead of perfect full-state measurements.

Another important point to highlight is that we do not presume that the central nervous system actually implements the linear regulator used in our model. The LQR design procedure was simply a tool to generate stabilizing controllers while simultaneously analyzing the influence of factors like the cost of control on balance performance.

## Conclusion

This study showed that a double inverted pendulum stabilized by a linear minimal-effort controller could account for the ground reaction force pattern observed in human quiet standing. Numerical simulations also informed the contribution of neural control and biomechanics in generating the pattern observed in human data, i.e. the frequency-dependence of the intersection point. The results suggest that the intersection point conveys quantitative information about human balance control strategies.

This study introduced a method to select optimal control and noise parameters that best reproduced human data. This method might be extended to study human neural control strategies in different contexts, e.g., balance in the frontal plane, balance on a beam, balance with and without assistive devices, or in other populations such as aged or neurologically impaired subjects.

## Figures and Tables

**Fig. 6:**
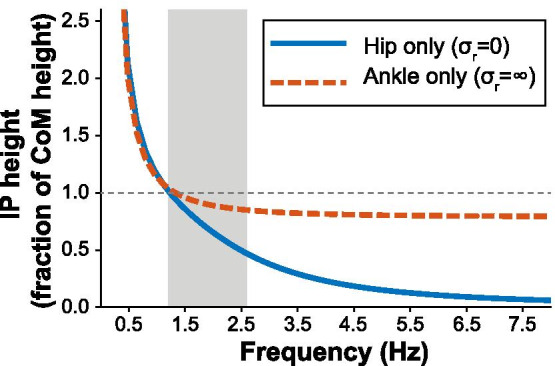
Height of the intersection point of the linearized double inverted pendulum model with two extreme $\sigma _r$ values: $\sigma _r= 0$ (hip noise only) and $\sigma _r = \infty$ (ankle noise only)

## Data Availability

Not applicable.

## Appendix 1: Intersection point of the single inverted pendulum

The intersection point below the center of mass at high frequencies observed in human data cannot be reproduced by a single inverted pendulum model. Consider a single inverted pendulum with mass *m*, center of mass position from the pivot $l_c$, moment of inertia about pivot $j'$, gravitational acceleration *g*, and actuated by ankle torque $\tau$. The equation of motion is

12 $$\begin{aligned} j'\ddot{q}-mg l_c \sin q = \tau = \tau _{\mathrm{{ctl}}}+w, \end{aligned}$$

where *q* is the angular displacement of the ankle joint with respect to the upright equilibrium posture, $\tau _{\mathrm{{ctl}}}$ is the control torque, and *w* is the additive actuation noise. For small motions typical of quiet standing, linearization of (12) is well justified:

$$\begin{aligned} j'\ddot{q}-mgl_cq = \tau . \end{aligned}$$

As introduced in (3), the intersection point is defined in terms of the orientation of the force, $\theta _F$, and the center of pressure,* CoP*:

$$\begin{aligned} \theta _F&= -\frac{F_x}{F_z} \approx \frac{mc\ddot{q}}{mg} = \frac{l_c}{g} \ddot{q}, \\ CoP&=\frac{\tau }{F_z} \approx \frac{\tau }{mg}=\frac{j'}{mg}\ddot{q}-l_c q. \end{aligned}$$

Taking the Laplace transform:

$$\begin{aligned} \Theta _F(s)&= \frac{l_c }{g}s^2Q(s), \\ COP(s)&= \left(\frac{j'}{mg}s^2-l_c \right)Q(s), \end{aligned}$$

where *s* is a complex variable, *Q*(*s*), $\Theta _F(s)$, and *COP*(*s*) are the Laplace transforms of *q*, $\theta _F$, and *CoP*, respectively. Denote $H(s)=Q(s)/W(s)$, the transfer function from input noise to output motion, where *W*(*s*) is the Laplace transform of *w*. To investigate the intersection point at high frequency, consider $s=i\Omega$ where $i^2=-1$ and $\Omega \rightarrow \infty$. As the first term of *COP*(*s*) dominates, $\Theta _F(s)$ and *COP*(*s*) have the same phase. Then, the variation of two output variables will be linear at high frequencies and the intersection point can be determined from the ratio of magnitudes of the two outputs:

$$\begin{aligned} \begin{aligned} IP(\Omega ) = \frac{\left|COP(i\Omega )\right|}{\left|\Theta _F (i\Omega )\right|} =\frac{\frac{j'}{mg}\Omega ^2+l_c}{\frac{l_c}{g}\Omega ^2} =\frac{j'\Omega ^2+mgl_c}{ml_c\Omega ^2}. \end{aligned} \end{aligned}$$

As $$\Omega \rightarrow \infty$$,

$$\begin{aligned} IP(\Omega ) \rightarrow \frac{j'}{ml_c} = \frac{j + ml_c^2}{ml_c^2} = l_c+ \frac{j}{ml_c}>l_c. \end{aligned}$$

Note that the centroidal moment of inertia is $j=j'-mc^2$. Therefore, the intersection point height must be greater than the center of mass height. The single inverted pendulum model cannot explain the intersection point behavior observed in human studies.

## Appendix 2: Nonlinear model equations

The equations of motion of the double inverted pendulum are expressed in (4). Note that the choice of generalized coordinates, $q_1$, $q_2$, are consistent with the generalized forces (torques) that are applied. The following details each component of the matrices in terms of the variables summarized in Table 1. $j'_1$ and $j'_2$ denote moments of inertia taken about ankle and hip joints, respectively. $l_{c1}$ is the distance from the ankle joint to the center of mass of link 1, and $l_{c2}$ is the distance from the hip joint to the center of mass of link 2. $\cos {(q_i)}$ and $\sin {(q_i)}$ are replaced with $c_i$ and $s_i$, respectively. Setting $\theta _2 = q_1 + q_2$, $\cos {(\theta _2)}$ and $\sin {(\theta _2)}$ are replaced with $c_{\theta _2}$ and $s_{\theta _2}$, respectively.

$$\begin{aligned} {\mathbf {M}}({\mathbf {q}})= & {} \begin{bmatrix} j'_1+j'_2+m_2(l_1^2+2l_1l_{c2}c_2) &{} j'_2+m_2l_1l_{c2}c_2\\ j'_2+m_2l_1l_{c2}c_2 &{} j'_2 \end{bmatrix} \\ \mathbf{C}(\mathbf{q},\dot{\mathbf{q}})= & {} m_2l_1l_{c2}s_2 \begin{bmatrix} -2\dot{q_2} &{} -\dot{q_2}\\ \dot{q_1} &{} 0 \end{bmatrix} \\ {\mathbf {G}}({\mathbf {q}})= & {} -g \begin{bmatrix} m_1l_{c1}s_1+m_2(l_1s_1+l_{c2}s_{\theta _2})\\ m_2l_{c2}s_{\theta _2} \end{bmatrix} \end{aligned}$$

The ground reaction forces are computed from the motion of the center of mass, ${\mathbf {r}}_{CoM}({\mathbf {q}})\in {\mathbb {R}}^{2\times 1}$. The acceleration of the center of mass was computed as

13 $$\begin{aligned} \ddot{{\mathbf {r}}}_{CoM} = [\ddot{r}_{CoM,x}, \ddot{r}_{CoM, z}]^T = \begin{bmatrix} \dot{{\mathbf {J}}}_{CoM}&{\mathbf {J}}_{CoM} \end{bmatrix} \dot{{\mathbf {x}}}. \end{aligned}$$

${\mathbf {J}}_{CoM}\in {\mathbb {R}}^{2\times 2}$ is the Jacobian of the center of mass with respect to the joint angles ${\mathbf {q}}$ and $\mathbf{x} = [{\mathbf{q}},\dot{\mathbf{q}}]^{T}$ is the state vector.

The Jacobian matrix and its derivative are given as follows:

$$\begin{aligned} {\mathbf {J}}_{CoM} = - \begin{bmatrix} {\mathbf {J}}_{CoM,1}&{\mathbf {J}}_{CoM,2} \end{bmatrix} \end{aligned}$$

and

$$\begin{aligned} \dot{{\mathbf {J}}}_{CoM} = \begin{bmatrix} {\dot{J}}_{CoM,(1,1)} &{} {\dot{J}}_{CoM,(1,2)}\\ {\dot{J}}_{CoM,(2,1)} &{} {\dot{J}}_{CoM,(2,2)} \end{bmatrix}, \end{aligned}$$

where

$$\begin{aligned} {\mathbf {J}}_{CoM,1}& = \begin{bmatrix} M_1l_{c1}c_1 + M_2(l_1c_1+l_{c2}c_{\theta _2})\\ M_1l_{c1}s_1 + M_2(l_1s_1+l_{c2}s_{\theta _2}) \end{bmatrix},\\ {\mathbf {J}}_{CoM,2}& = \begin{bmatrix} M_2l_{c2}c_{\theta _2}\\ M_2l_{c2}s_{\theta _2} \end{bmatrix}, \end{aligned}$$

$$\begin{aligned} {\dot{J}}_{CoM,(1,1)}& = M_1l_{c1}\dot{q_1}s_1 + M_2(l_1\dot{q_1}s_1+l_{c2}\dot{\theta _2}s_{\theta _2}),\\ {\dot{J}}_{CoM,(1,2)}& = M_2l_{c2}\dot{\theta _2}s_{\theta _2},\\ {\dot{J}}_{CoM,(2,1)}& = -M_1l_{c1}\dot{q_1}c_1 - M_2(l_1\dot{q_1}c_1+l_{c2}\dot{\theta _2}c_{\theta _2}),\\ {\dot{J}}_{CoM,(2,2)}&= -M_2l_{c2}\dot{\theta _2}c_{\theta _2}, \end{aligned}$$

and $$M_1 = \frac{m_1}{m_1+m_2}$$ and $$M_2 = \frac{m_2}{m_1+m_2}$$.

## Appendix 3: Linearized state-space matrices

Linearizing the equations of motion about the stable upright position, we are left with (8). The state-space matrices are

$$\begin{aligned} {\mathbf {A}}_{lin}= & {} \begin{bmatrix} {\mathbf {0}} &{} {\mathbf {I}}\\ {\mathbf {M}}^{-1}\frac{\partial {\mathbf {G}}}{\partial {\mathbf {q}}} &{} {\mathbf {0}} \end{bmatrix}_{{\mathbf {x}} = {\mathbf {x}}^*, {\varvec{\tau }} = {\varvec{\tau }}^*} \\ {\mathbf {B}}_{lin}= & {} \begin{bmatrix} {\mathbf {0}}\\ {\mathbf {M}}^{-1}{\mathbf {B}} \end{bmatrix}_{{\mathbf {x}} = {\mathbf {x}}^*, {\varvec{\tau }} = {\varvec{\tau }}^*}, \end{aligned}$$

where

$$\begin{aligned} \frac{\partial {\mathbf {G}}}{\partial {\mathbf {q}}}\bigg \vert _{{\mathbf {x}} = {\mathbf {x}}^{*}}= -g \begin{bmatrix} m_1l_{c1} + m_2(l_1+l_{c2}) &{} m_2l_{c2}\\ m_2l_{c2} &{} m_2l_{c2} \end{bmatrix} \end{aligned}$$

and $${\mathbf {B}} = {\mathbf {I}}_2$$.

## Appendix 4: Intersection point of the linearized double inverted pendulum

Noting (1) and (7), consider two outputs ${\mathbf {y}}=[y_1, y_2]^T$:

$$y_1=-F_x=-m\ddot{r}_{CoM,x}, \quad y_2=\tau _1.$$

From (13),

$$\begin{aligned} y_1 &= -m[1, 0]\ddot{\mathbf{r}}_{CoM} \\ &= -m[1, 0][\dot{\mathbf{J}}_{CoM},\mathbf{J}_{CoM}]\dot{\mathbf{x}} \\&\triangleq \mathbf{J}_{y_1}\dot{\mathbf{x}}. \end{aligned} $$

Then, linearized output equations can be obtained as

$$\begin{aligned} {\mathbf {y}}=\mathbf {Cx+D}{\varvec{\tau }} = \begin{bmatrix} {\mathbf {C}}_1\\ {\mathbf {C}}_2 \end{bmatrix} {\mathbf {x}} + \begin{bmatrix} {\mathbf {D}}_1\\ {\mathbf {D}}_2 \end{bmatrix}\varvec{\tau }, \end{aligned}$$

where ${\mathbf {C}}_1 = {\mathbf {J}}_{y_1} {\mathbf {A}}_{lin} {\text{and}} {\mathbf {D}}_1 = {\mathbf {J}}_{y_1}{\mathbf {B}}_{lin}$, evaluated at $({{\mathbf {x}}, \varvec{\tau })=({\mathbf {x}}^*, \varvec{\tau }^*})$, and ${\mathbf {C}}_2 = {\mathbf {0}} {\text{and}} {\mathbf {D}}_2 = [1, 0]$. With controller ${\varvec{\tau }}=-\mathbf {Kx+w}$ as in (6) and (10), the closed-loop linear system can be constructed as

14 $$\begin{aligned} {\left\{ \begin{array}{ll} {{\dot{\mathbf{x}}} = ({\mathbf {A}}_{lin}-{\mathbf {B}}_{lin}{\mathbf {K}}){\mathbf {x}}+{\mathbf {B}}_{lin}{\mathbf {w}} = {\mathbf {A}}_{\mathrm{{cl}}}{\mathbf {x}}+{\mathbf {B}}_{lin}{\mathbf {w}}}\\ y_1 = ({\mathbf {C}}_1-{\mathbf {D}}_1{\mathbf {K}}){\mathbf {x}} +{\mathbf {D}}_1{\mathbf {w}} = {\mathbf {C}}_{\mathrm{{cl,1}}}{\mathbf {x}} +{\mathbf {D}}_1{\mathbf {w}}\\ y_2 = ({\mathbf {C}}_2-{\mathbf {D}}_2{\mathbf {K}}){\mathbf {x}}+{\mathbf {D}}_2{\mathbf {w}} = {\mathbf {C}}_{\mathrm{{cl,2}}}{\mathbf {x}}+{\mathbf {D}}_2{\mathbf {w}}. \end{array}\right. } \end{aligned} $$

The multi-input, multi-output (MIMO) transfer function can be obtained

$$\begin{aligned} {\mathbf {Y}}(s) = {\mathbf {H}}(s){\mathbf {W}}(s), \quad {\mathbf {H}}(s)= \begin{bmatrix} H_{11}(s) &{} H_{12}(s)\\ H_{21}(s) &{} H_{22}(s) \end{bmatrix} \end{aligned}$$

where ${\mathbf {Y}}(s)$ and ${\mathbf {W}}(s)$ are the Laplace transforms of ${\mathbf {y}}$ and ${\mathbf {w}}$, respectively. The intersection point at each frequency can be obtained by the procedure outlined in the  Methods section, as the inverse of the slope of the principal eigenvector. If $y_1(t)$ and $y_2(t)$ are harmonic, this procedure is equivalent to finding the slope of the major axis of the ellipsoid that the two signals form.

Assuming two harmonic signals $y_i(t)$ with magnitude $\nu _i$ and phase $\phi _i$ at frequency $\Omega$,

$$\begin{aligned} y_1 (t) = \nu _1\sin (\Omega t + \phi _1), \quad y_2 (t) = \nu _2\sin (\Omega t + \phi _2), \end{aligned}$$

an implicit formula for the ellipsoid can be written in quadratic form,

$$\begin{aligned} \sin ^2\phi = \left[ y_2, y_1\right] \left[ \begin{array}{cc} \frac{1}{\nu _2^2} &{} -\frac{\cos {\phi }}{\nu _1\nu _2}\\ -\frac{\cos {\phi }}{\nu _1\nu _2} &{} \frac{1}{\nu _1^2}\\ \end{array} \right] \left[ \begin{array}{c} y_1\\ y_2\\ \end{array} \right] \end{aligned}$$

where $\phi =\phi _1-\phi _2$. The eigenvector corresponding to the smaller eigenvalue is the major axis and its slope is the inverse of the intersection point as in Fig. 2.

Consider two extreme cases where ankle noise is zero ($w_1=0$ and $\sigma _r = 0$) and hip noise is zero ($w_2=0$ and $\sigma _r = \infty$). For example, when hip noise is zero, substituting $s = i\Omega$,

$$\begin{aligned} \frac{y_1}{w_1}\left( i\Omega \right) =H_{11}\left( i\Omega \right) , \frac{y_2}{w_1}\left( i\Omega \right) =H_{21}\left( i\Omega \right) \end{aligned}$$

and

$$\begin{aligned}&\nu _1=\left| H_{11}\left( i\Omega \right) \right| , \phi _1=\angle H_{11} \left( i\Omega \right) , \\&\nu _2=\left| H_{21}\left( i\Omega \right) \right| ,\phi _2=\angle H_{21}\left( i\Omega \right) . \end{aligned}$$

The intersection point height can be calculated using this method at different frequencies as shown in Fig. 6. Since we are examining a linearized model, non-zero ankle and hip noise responses would be some combination of these two extreme responses.

## Abbreviations

LQR: Linear quadratic regulator
CoP: Center of pressure
IP: Intersection point
CoM: Center of mass

## Footnotes

1 To ensure stability, the state-space matrices ${\mathbf {A}}_{lin}$ and ${\mathbf {B}}_{lin}$ must be a controllable pair, the ${\mathbf {Q}}$ matrix must be symmetric positive semi-definite, and the ${\mathbf {R}}$ matrix must be symmetric positive definite.
